# Phagocytosis of microparticles increases responsiveness of macrophage-like cell lines U937 and THP-1 to bacterial lipopolysaccharide and lipopeptide

**DOI:** 10.1038/s41598-021-86202-5

**Published:** 2021-03-24

**Authors:** Takayuki Ueno, Yumi Yamamoto, Kiyoshi Kawasaki

**Affiliations:** Faculty of Pharmaceutical Sciences, Doshisha Women’s College of Liberal Arts Kodo, Kyotanabe, Kyoto Japan

**Keywords:** Biochemistry, Cell biology, Immunology

## Abstract

Following bacterial infection, macrophages produce pro-inflammatory cytokines in response to bacterial cell components, including lipopolysaccharide (LPS) and lipopeptide, and simultaneously phagocytize and digest the invading bacteria. To study the effects of phagocytosis on pro-inflammatory responses, we determined if phagocytosis of polystyrene latex beads with ~ 1 µm diameter increases pro-inflammatory cytokine expression by human macrophage-like U937 and THP-1 cells stimulated with LPS. Treating macrophage-like cells with beads coated with IgG to facilitate Fcγ receptor-mediated phagocytosis increased LPS-induced expression of pro-inflammatory cytokines, including tumor necrosis factor-alpha, interleukin-1 beta, and interleukin-6. Treatment with beads coated with poly-l-lysine to facilitate Fcγ receptor–independent phagocytosis also increased LPS-induced cytokine expression. Our results indicate that LPS-induced pro-inflammatory responses are enhanced by bead phagocytosis regardless of the uptake mechanism. Additionally, phagocytosis enhanced LPS-induced NF-κB activation, suggesting that Toll-like receptor (TLR) 4 signaling is enhanced by phagocytosis. Furthermore, bead phagocytosis enhanced pro-inflammatory responses in U937 cells stimulated with lipopeptide, a ligand for the TLR2/TLR6 heterodimeric receptor. In conclusion, microparticle phagocytosis by macrophage-like U937 and THP-1 cells enhances the innate immune response induced by bacterial components.

## Introduction

Macrophages recognize various bacterial components, including lipopolysaccharide (LPS) and lipopeptide, using pattern recognition receptors, including Toll-like receptors (TLRs)^1–4^. The recognition of bacterial components by pattern recognition receptors induces cellular signaling essential for innate immune responses, including the expression of pro-inflammatory cytokines, such as tumor necrosis factor-α (TNFα), interleukin-1β (IL-1β), and interleukin-6 (IL-6)^1–4^. In contrast, overexpression of pro-inflammatory cytokines can cause severe inflammation, leading to tissue damage and ultimately, death^5–7^. Therefore, a tightly controlled pro-inflammatory response is important for host defense against bacterial infection.

In addition to the pro-inflammatory responses induced by bacterial components, macrophages phagocytize and digest bacteria upon infection. Phagocytosis of bacteria is triggered by the binding of bacteria to phagocytic cell surface receptors^8–11^. Newly formed phagosomes become increasingly acidic as they mature, and eventually fuse with lysosomes to form phagolysosomes that degrade bacteria^9–12^. Liberation, degradation, and condensation of bacterial components within phagosomes and phagolysosomes should affect pro-inflammatory responses in macrophages^13^. The formation of phagosomes containing *Escherichia coli* causes a much more robust expression of interferon-β by human monocytes compared to stimulation with LPS alone^14^. Innate immune receptors, including TLR4 and TLR2/6 heterodimeric receptors, which can sense bacterial components at the cell surface, are recruited to phagosomes following phagocytosis^15–17^.

The reduced size and closed surface of phagosomes and phagolysosomes increase the concentration of their ligands, which are liberated from bacteria, and the increased concentration may fully activate the receptors^11,18^. Although the importance of phagosomes and phagolysosomes upon recognition of bacterial components for initiating efficient pro-inflammatory responses has become increasingly apparent, the regulatory effects of phagocytosis itself on cytokine expression induced by bacterial components remains to be determined. In this study, we determined the effects of phagocytosis itself on pro-inflammatory cytokine expression by bacterial components in human macrophage-like cell lines. To study the effects of phagocytosis on innate immune responses, we used polystyrene latex beads, which are microparticles comparable in size to bacteria but are hardly degraded in a cellular environment^19^. Moreover, such beads do not contain bacterial components that activate innate immune receptors.

## Results

### Phagocytosis of beads mediated by Fcγ receptor enhances LPS-induced pro-inflammatory responses in human macrophage-like cells

**Figure 1:**
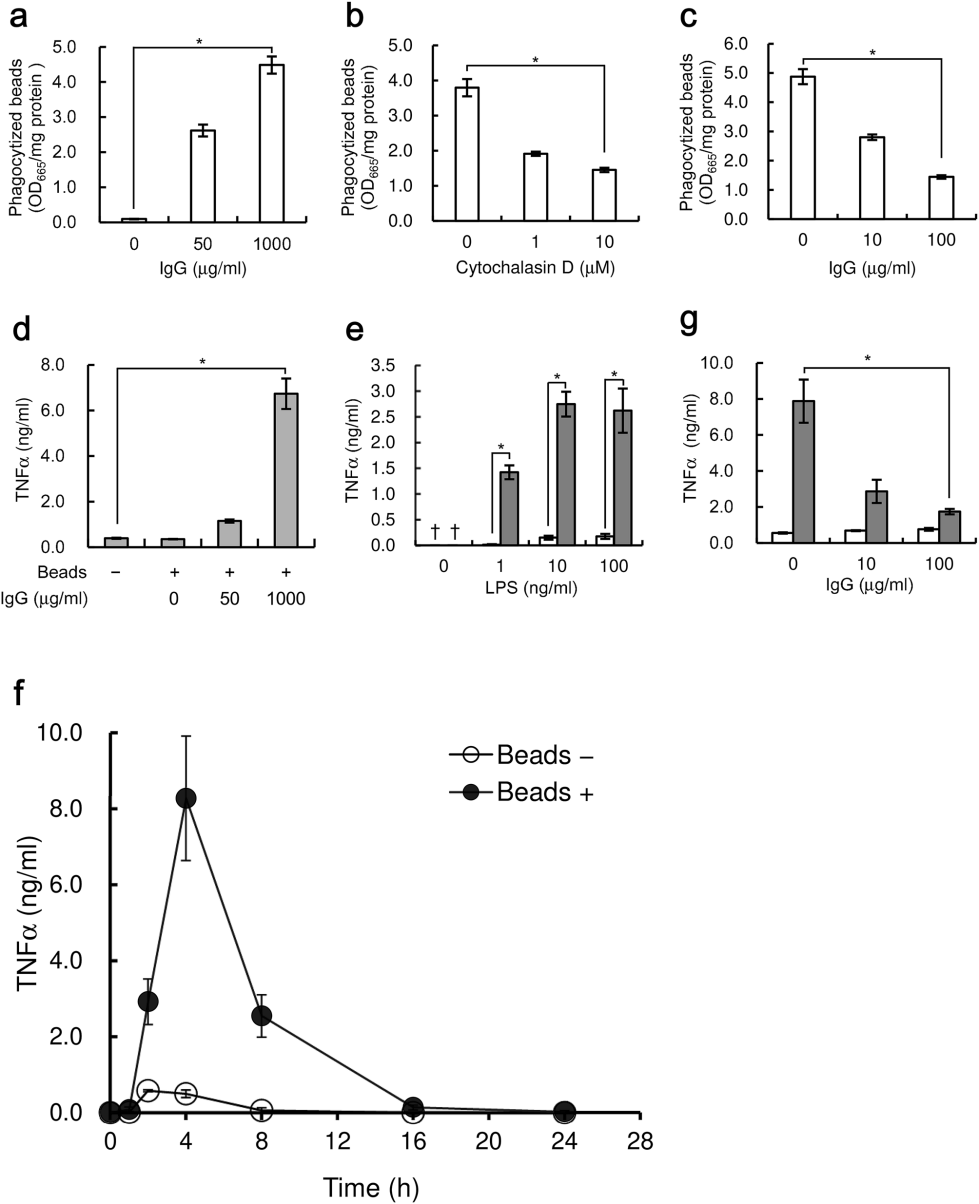
Phagocytosis of IgG-coated beads increases LPS-induced TNFα expression from U937 cells. Beads were added to U937 cells at a bead-to-cell ratio of 500. The number of phagocytized beads **(a**–**c)** or the amount of TNFα in culture supernatant **(d**–**g)** was determined. Daggers (†) indicate values below the detection limit. The data shown are mean values with standard deviations derived from triplicate measurements. Data were analyzed by the t-test. *P < 0.05. **(a)** Cells were cultured with beads pre-treated with the indicated concentrations of human IgG for 4 h. (**b**) Cells were cultured with indicated concentrations of Cytochalasin D for 1 h. Next, the beads pre-treated with 1000 µg/ml IgG were added to the culture and cultured for an additional 4 h. **(c)** Cells were cultured with the indicated concentrations of human IgG for 4 h. The beads pre-treated with 1000 µg/ml IgG were added to the culture and the cells were cultured for an additional 4 h. **(d)** Cells stimulated with 100 ng/ml LPS were cultured simultaneously either with ( +) or without (−) beads pre-treated with the indicated concentrations of human IgG. **(e)** Cells stimulated with indicated concentrations of LPS were cultured simultaneously with (gray bar) or without (white bar) beads pre-treated with 1000 µg/ml human IgG. **(f)** Cells stimulated with 10 ng/ml LPS were cultured simultaneously with (closed circle, Beads +) or without (open circle, Beads −) beads pre-treated with 1000 µg/ml human IgG for 0, 1, 2, 4, 8, 16, and 24 h. **(g)** Cells were cultured with indicated concentrations of human IgG for 4 h prior to addition of LPS and beads. Next, cells stimulated with 10 ng/ml LPS were cultured simultaneously either with (gray bar) or without (white bar) beads pre-treated with 1000 µg/ml human IgG.

Macrophages phagocytize foreign substances, including bacteria and micro artificial particles such as polystyrene latex beads. Phagocytosis proceeds through many pathways, and one major pathway is Fc*γ* receptor-dependent phagocytosis^8–10^. Thus, we investigated the conditions under which human macrophage-like U937 cells efficiently phagocytize micro beads using the Fcγ receptor. Polystyrene latex beads with diameters of approximately 0.8 µm, which mimic the size of bacteria, were pre-treated with human IgG, and the efficiency of their phagocytosis by U937 cells was measured. Pre-treating beads with 1000 µg/ml human IgG caused an approximately 50-fold increase in phagocytosis compared with untreated beads (Fig. 1a). Under these conditions, 121 beads/cell were estimated to be phagocytized. Inhibiting actin polymerization in cells with 10 µM cytochalasin D decreased phagocytosis of beads by 62% (Fig. 1b). In addition, blocking Fcγ receptor through pre-treatment of U937 cells with 100 µg/ml human IgG decreased phagocytosis by 71% (Fig. 1c). These results, taken together, indicate that U937 cells efficiently phagocytized polystyrene latex beads pre-treated with IgG through recognition by the Fcγ receptor.

Thus, we examined the effect of bead phagocytosis on TNFα expression by LPS-stimulated U937 cells. Treating the cells with IgG-coated beads increased TNFα expression by LPS-stimulated cells compared to those without beads (Fig. 1d,e), and TNFα production increased depending on the amount of phagocytized beads (Fig. 1a,d). LPS-induced TNFα expression nearly reached a maximum in the presence of 10 ng/ml LPS, and it is noteworthy that bead phagocytosis caused an approximately 17-fold increase in maximum TNFα expression induced by LPS stimulation alone (Fig. 1e). Bead treatment alone did not induce TNFα expression, indicating that phagocytosis itself does not induce TNFα expression (Fig. 1e). TNFα expression induced by LPS reached a maximum at 2 h after LPS stimulation, and subsequently decreased (Fig. 1f). Phagocytosis of beads increased TNFα expression induced by LPS, and TNFα expression reached a maximum at 4 h after stimulation (Fig. 1f). To further confirm the causal relationship between phagocytosis and LPS-induced TNFα expression, we determined the effect of inhibiting phagocytosis. Inhibiting phagocytosis by pre-treating cells with 100 µg/ml IgG attenuated the increase in TNFα expression by 78% (Fig. 1g). IgG pre-treatment of cells did not inhibit TNFα expression in the absence of beads, indicating that IgG did not inhibit LPS-induced TNFα expression (Fig. 1g). Taken together, these results indicate that polystyrene latex beads phagocytized through the Fcγ receptor increases TNFα expression in U937 cells stimulated with LPS.

**Figure 2:**
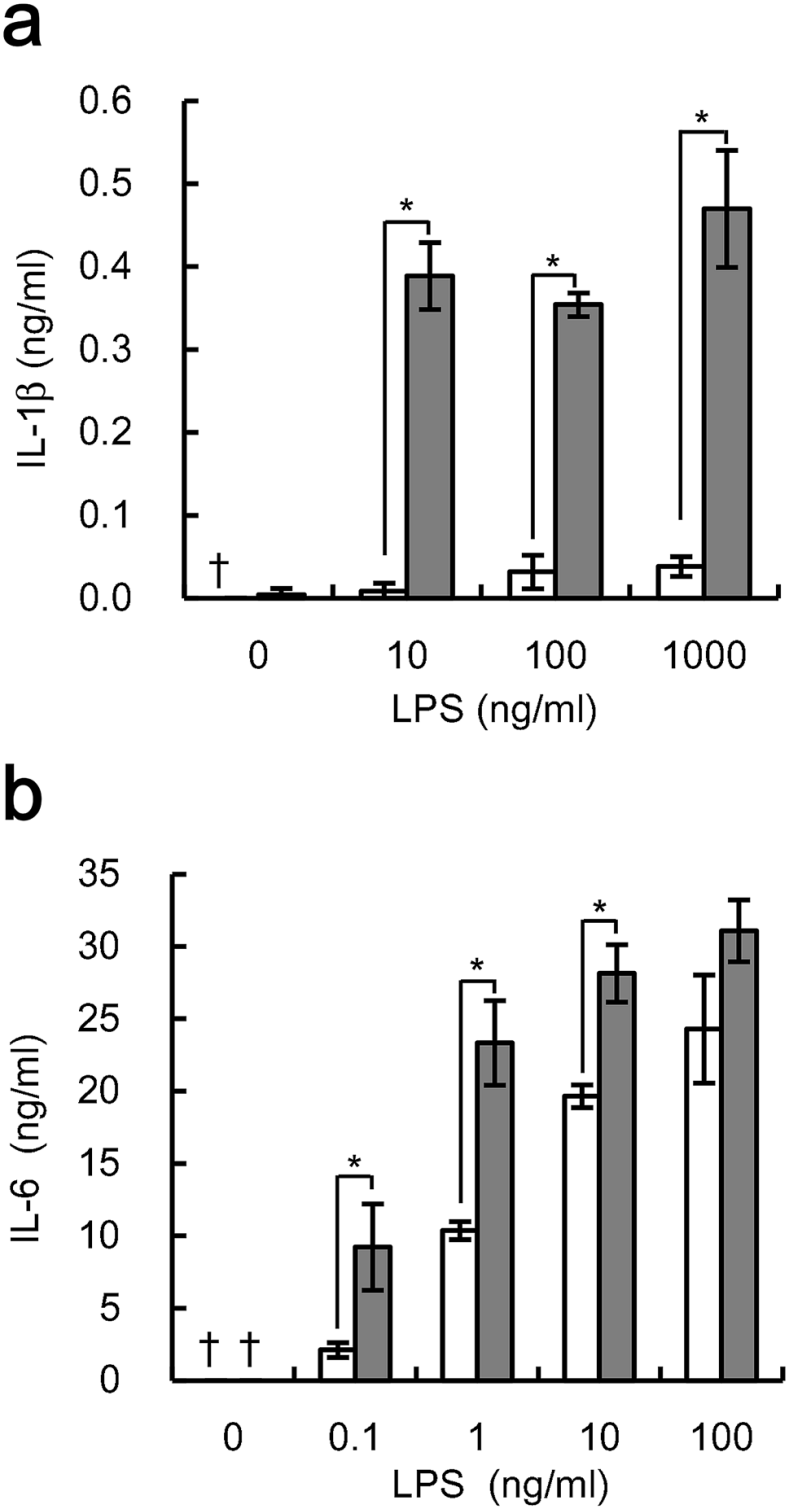
Phagocytosis of IgG-coated beads increases LPS-induced expression of IL-1β and IL-6 by U937 cells**.** U937 cells stimulated with indicated concentrations of LPS were cultured simultaneously either with (gray bar) or without (white bar) beads pre-treated with 1000 µg/ml human IgG at a ratio of 500 beads/cell. After cultivation, the amounts of IL-1β **(a)** and IL-6 **(b)** were determined. The data shown are mean values with standard deviations derived from triplicate measurements. Daggers (†) indicate below the detection limit. Data were analyzed by the t-test. *P < 0.05.

Furthermore, we determined the effects of phagocytosis on LPS-induced expression of IL-1β and IL-6. Phagocytosis of IgG-coated beads also increased both IL-1β and IL-6 expression at all LPS concentrations tested (Fig. 2). LPS-induced expression of IL-1β and IL-6 reached nearly maximum levels at 100 ng/ml LPS. Phagocytosis increased IL-1β expression by approximately 12 times, compared to the maximum level (Fig. 2). In contrast, the rate of increase of IL-6 expression was lower than that of either TNFα or IL-1β expression. Treatment with the beads alone did not induce either IL-1β or IL-6 expression (Fig. 2). Together, these results indicate that polystyrene latex beads are phagocytosed through Fcγ receptor-enhanced, LPS-induced pro-inflammatory responses in U937 cells.

**Figure 3:**
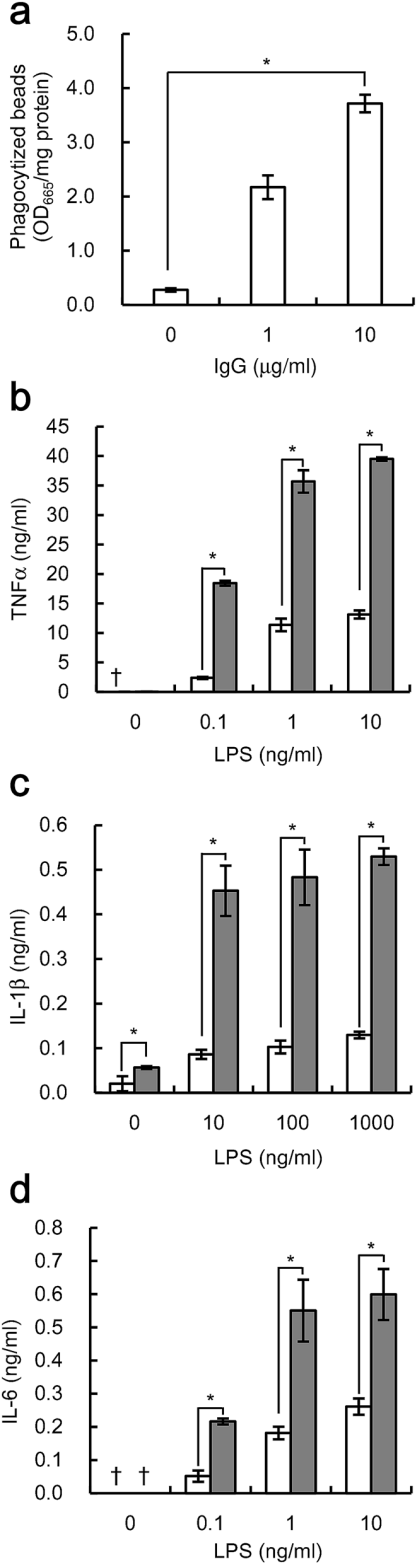
Phagocytosis of IgG-coated beads increases LPS-induced pro-inflammatory cytokine expression in THP-1 cells. **(a)** THP-1 cells were cultured with beads pre-treated with the indicated concentrations of human IgG for 4 h at a ratio of 500 beads/cell. The numbers of phagocytized beads were evaluated. Data shown are mean values with standard deviations derived from triplicate measurements. **(b**–**d)** THP-1 cells stimulated with indicated concentrations of LPS were cultured simultaneously either with (gray bar) or without (white bar) beads pre-treated with 10 µg/ml IgG at a ratio of 500 beads/cell. After culturing, the amounts of TNFα **(b)**, IL-1β **(c)**, and IL-6 **(d)** were determined. Data shown are mean values with standard deviations derived from triplicate measurements. Daggers (†) indicate below the detection limit. Data were analyzed by the t-test. *P < 0.05.

We also investigated whether the enhancement of the pro-inflammatory response was observed in human macrophage-like THP-1 cells. Pre-treating the beads with 10 µg/ml human IgG caused an approximately 13-fold increase in phagocytosis (Fig. 3a), and we used the IgG-treated beads for further analysis. Bead treatment increased TNFα, IL-1β, and IL-6 expression in THP-1 cells stimulated with LPS; THP-1 cells stimulated with 10 ng/ml LPS showed 3.0-fold, 5.3-fold, and 2.3-fold increases in TNFα, IL-1β, and IL-6 expression, respectively. The expression levels measured were beyond the maximum levels achieved by LPS stimulation alone (Fig. 3b–d). Taken together, Fcγ receptor-mediated phagocytosis enhances LPS-induced pro-inflammatory responses in human macrophage-like cells.

### *Fc*γ* receptor-independent phagocytosis also enhances LPS-induced pro-inflammatory responses*

**Figure 4:**
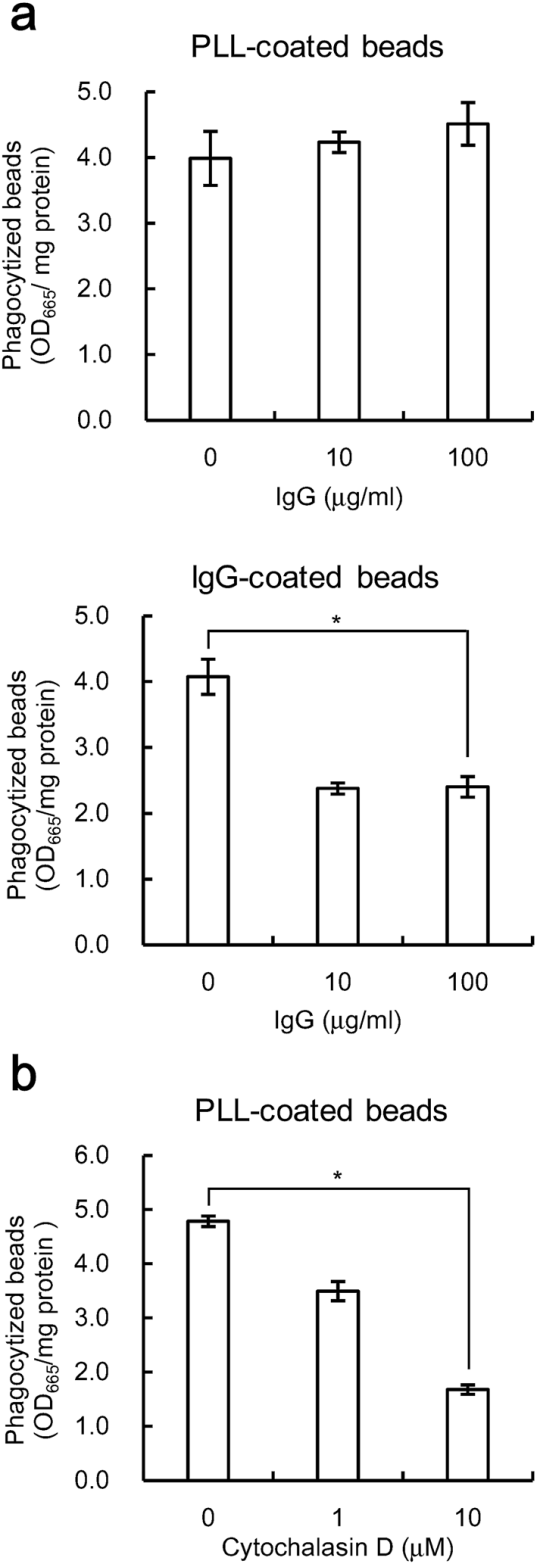
Poly-L-lysine (PLL)-coated beads are phagocytized independently of Fcγ receptor. The numbers of beads phagocytized were evaluated. The data shown are the mean values with standard deviations derived from triplicate measurements. Data were analyzed by the t-test. *P < 0.05. **(a)** Cells were cultured with the indicated concentrations of human IgG for 4 h, and were further cultured with beads pre-treated with either PLL or 10 μg/ml human IgG at 500 beads/cell for 1 h. **(b)** Cells were cultured with the indicated concentrations of cytochalasin D added for 1 h before being cultured with PLL-coupled beads for 4 h at a ratio of 500 beads/cell.

To further investigate the effects of phagocytosis on cytokine expression, polystyrene latex beads with an approximate diameter of 1 µm were covalently coupled to poly-L-lysine (PLL) before being used for this study. Positively charged particles, including PLL-coated beads, are known to be efficiently phagocytized by macrophages independently of the Fcγ receptor^20^. Phagocytosis of PLL-coated beads was observed by THP-1 cells; we found that phagocytosis of PLL-coated beads by THP-1 cells (Fig. 4a) was much greater than phagocytosis of uncoated beads (Fig. 3a). In contrast, PLL-dependent phagocytosis was not observed in U937 cells (data not shown). We therefore used THP-1 cells for further analysis.

**Figure 5:**
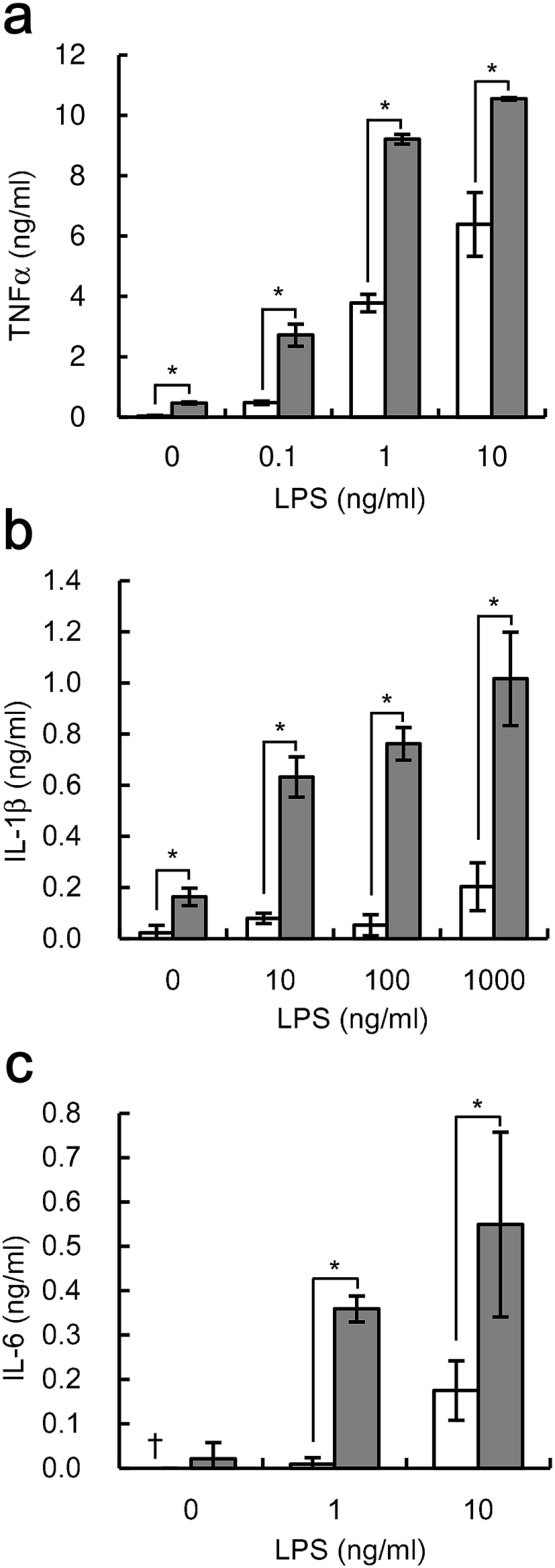
Phagocytosis of PLL-coated beads increases LPS-induced pro-inflammatory cytokine expression**.** THP-1 cells stimulated with indicated concentrations of LPS were cultured simultaneously with (gray bar) or without (white bar) beads coupled to PLL at a ratio of 500 beads/cell. After culture, expression of TNFα **(a)**, IL-1β **(b)**, and IL-6 **(c)** were determined. Data shown are mean values with standard deviations derived from triplicate measurements. A dagger (†) indicates values below the detection limit. Data were analyzed by the t-test. *P < 0.05.

Pre-treating THP-1 cells with 100 µg/ml IgG inhibited phagocytosis of IgG-coated beads by 42% but not PLL-coated beads (Fig. 4a). In contrast, pre-treatment of THP-1 cells with 10 µM cytochalasin D inhibited phagocytosis of PLL-coated beads by 65% (Fig. 4b). These results indicate that phagocytosis of PLL-coated beads is not mediated by the Fcγ receptor. Thus, we determined whether phagocytosis of PLL-coated beads affects cytokine expression by LPS-stimulated THP-1 cells. Phagocytosis of PLL-coated beads increased TNFα, IL-1β, and IL-6 expression in cells stimulated with 10 ng/ml LPS by 1.7-fold, 8.0-fold, and 3.1-fold, respectively (Fig. 5). Phagocytosis of PLL-coated beads alone slightly increased the cytokine expression, but the increments were much lower than those with LPS-stimulated cells (Fig. 5). These results indicate that Fcγ receptor-independent phagocytosis also enhances LPS-induced pro-inflammatory responses in THP-1 cells.

### Phagocytosis enhances LPS-induced activation of NF-κB

**Figure 6:**
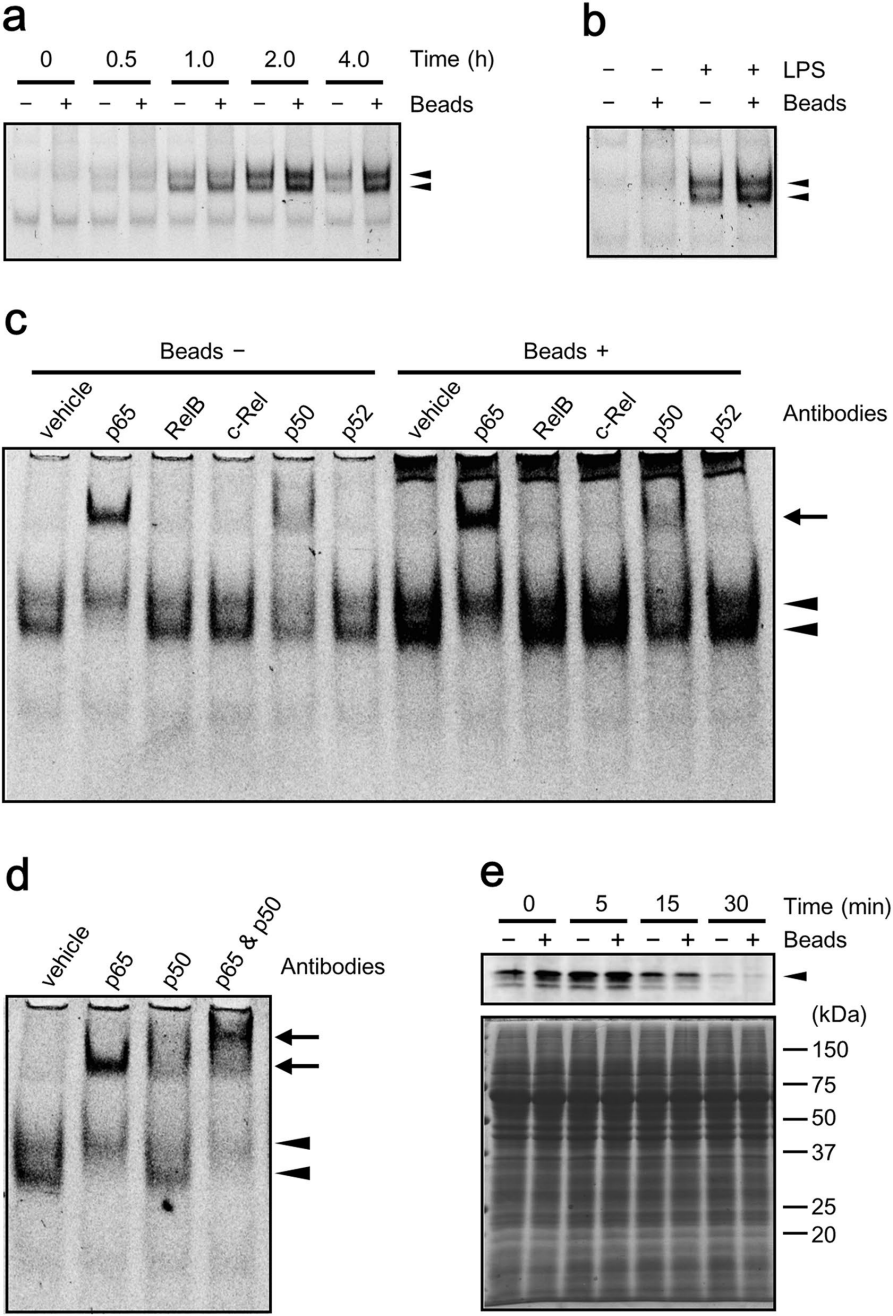
Activation of NF-κB induced by LPS is enhanced by phagocytosis of IgG-coated beads. **(a,b)** Activation of NF-κB in U937 cells was examined by EMSA. Arrowheads indicate the mobility-shifted bands. Full-length gels are presented in Supplementary Fig. 1. Cells stimulated with 0.1 ng/ml LPS were cultured simultaneously either with ( +) or without (−) beads pre-treated with 1000 µg/ml human IgG at a ratio of 1000 beads/cell for the indicated duration **(a)**. Cells stimulated with ( +) or without (−) 1 ng/ml LPS were cultured simultaneously either with ( +) or without (−) beads pre-treated with 1000 µg/ml human IgG at a ratio of 1000 beads/cell for 2 h **(b)**. **(c,d)** Binding of the NF-κB complex with indicated antibodies was examined by supershift assay**.** Arrowheads indicate the mobility-shifted bands and arrows indicate supershifted bands. Full-length gels are presented in Supplementary Fig. 1. Cells stimulated with 0.1 ng/ml LPS were cultured simultaneously either with (Beads +) or without (Beads −) beads pre-treated with 1000 µg/ml human IgG at a ratio of 1000 beads/cell for 2 h **(c)**. Cells were stimulated with 0.1 ng/ml LPS for 2 h **(d)**. **(e)** Degradation of IκB-α was examined by western blot (upper panel). Proteins were analyzed by Coomassie brilliant blue staining (lower panel). An arrowhead indicates the IκB-α bands. A full-length blot is presented in Supplementary Fig. 1. Cells stimulated with 50 ng/ml LPS were cultured simultaneously either with (+) or without (−) beads pre-treated with 1000 µg/ml human IgG at a ratio of 2,000 beads/cell for the indicated duration.

We found that bead phagocytosis enhances the expression of pro-inflammatory cytokines from macrophage-like U937 and THP-1 cells stimulated with LPS. In order to determine whether enhancement occurred at the level of transcription or post-transcriptionally, we examined the effects of phagocytosis of beads on NF-κB activation in U937 cells. LPS-induced activation of NF-κB, which was represented by two mobility-shifted bands^21^, was observed 1 h after stimulation, reached a maximum level at 2 h after stimulation, and decreased at 4 h after stimulation (Fig. 6a). Phagocytosis of IgG-coated beads increased NF-κB activation at all of these time points, and NF-κB activation reached a maximum level at 2 h and lasted up to 4 h thereafter (Fig. 6a). The delayed peaks of NF-κB due to phagocytosis were consistent with enhanced TNFα expression after phagocytosis (Fig. 1f). In addition, phagocytosis of IgG-coated beads alone did not activate NF-κB (Fig. 6b). These results indicate that phagocytosis enhances LPS-induced NF-κB activation in U937 cells.

To examine the effect of phagocytosis on the composition of the NF-κB complex, binding of the NF-κB complex was analyzed with antibodies against NF-κB subunits. Anti-p65 antibody and anti-p50 antibody shifted NF-κB bands activated by LPS but this did not occur with any other antibodies (Fig. 6c). Phagocytosis increased the shifted bands by anti-p65 as well as anti-p50 antibodies, and it did not induce any band shifts when probed with other antibodies including anti-RelB, anti-c-Rel, and anti-p52 antibodies, suggesting that phagocytosis did not change the composition of the NF-κB complex (Fig. 6c). Binding of the NF-κB complex simultaneously with both anti-p65 and anti-p50 antibodies further shifted the NF-κB band, confirming the presence of the p65/p50 heterodimer in the NF-κB complex (Fig. 6d). These results, taken together, indicated that phagocytosis increased LPS-induced activation of p65/p50. Furthermore, we examined the effect of phagocytosis on degradation of IκB-α. IκB-α in cytoplasm decreased 15 min after LPS stimulation and almost disappeared 30 min after stimulation (Fig. 6e). IκB-α levels at these time points were comparable between cells that phagocytized beads and those that did not, indicating that phagocytosis did not promote the degradation of IκB-α (Fig. 6e).

### TLR2/6-mediated pro-inflammatory responses are also enhanced by phagocytosis

**Figure 7:**
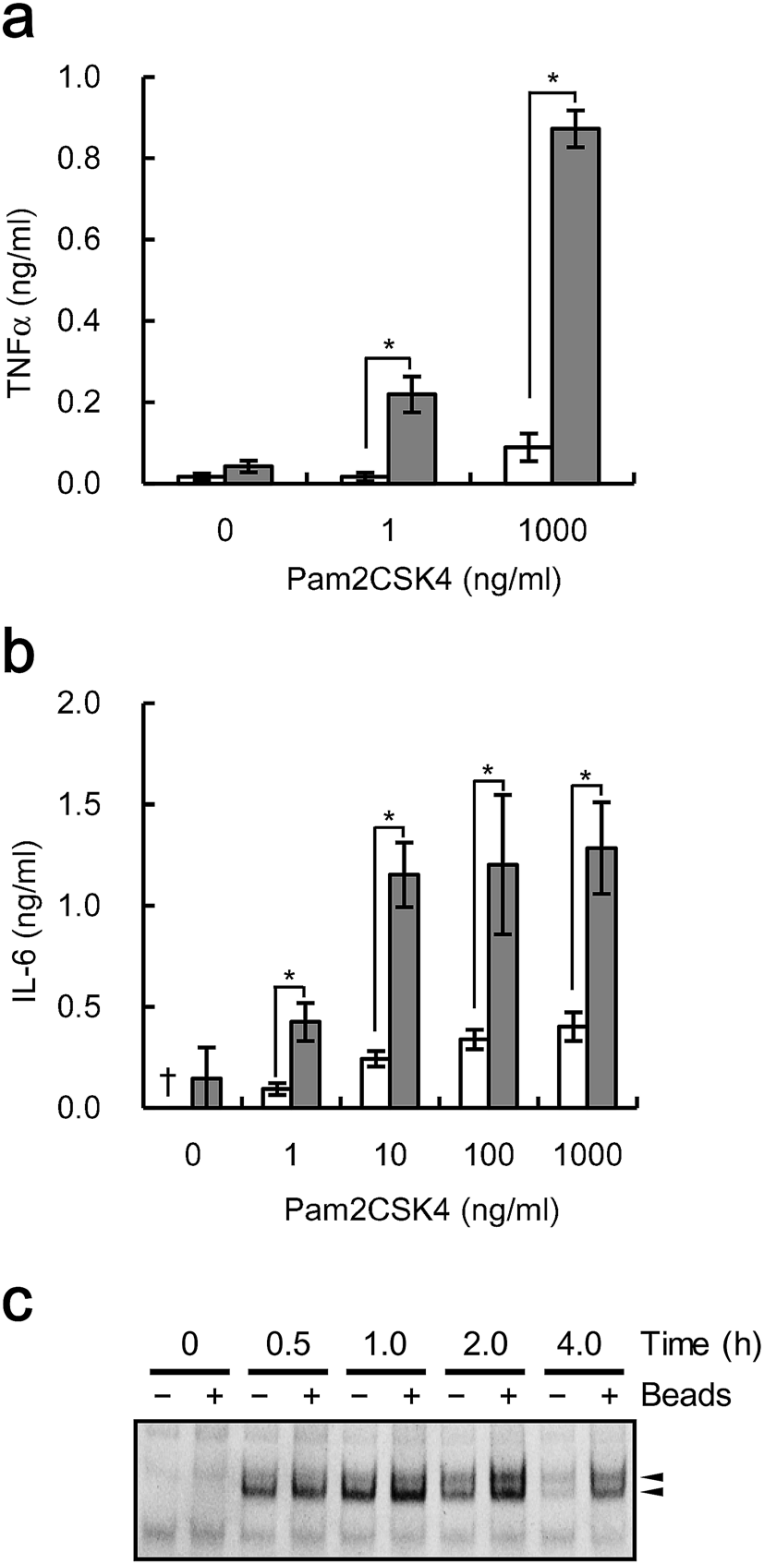
Pam2CSK4-induced pro-inflammatory cytokine expression and activation of NF-κB are enhanced by phagocytosis of IgG-coated beads. **(a,b)** U937 cells stimulated with the indicated concentrations of Pam2CSK4 were cultured simultaneously either with (gray bar) or without (white bar) beads pre-treated with 1000 µg/ml human IgG at a ratio of 500 beads/cell. After culturing, TNFα **(a)** and IL-6 **(b)** expression levels were determined. The data shown are the mean values with standard deviations derived from triplicate measurements. A dagger (†) indicates values below the detection limit. Data were analyzed by the t-test. *P < 0.05. **(c)** U937 cells stimulated with 1 ng/ml Pam2CSK4 were cultured simultaneously either with (+) or without (−) beads pre-treated with 1000 µg/ml human IgG at a ratio of 1000 beads/cell for the indicated duration, and NF-κB activation was examined by EMSA. Arrowheads indicate shifted bands. A full-length gel is presented in Supplementary Fig. 1.

In order to determine whether the effects of phagocytosis are limited to stimulation with LPS, we sought to determine the effects of phagocytosis on macrophage-like cells stimulated with the synthetic diacyl lipopeptide, Palmitoyl-2-cysteine-serine-lysine-4 (Pam2CSK4), which is a ligand for the TLR2/6 heterodimeric receptor^22^. U937 cells stimulated with Pam2CSK4 were treated with IgG-coated beads, and expression of both TNFα and IL-6 was measured. Enhancement of cytokine expression by phagocytosis was observed; a 9.8-fold increase in TNFα expression and a 3.2-fold increase in IL-6 expression were observed from cells stimulated with 1000 ng/ml Pam2CSK4 (Fig. 7a,b). In addition, phagocytosis of IgG-coated beads increased NF-κB activation in U937 cells stimulated with Pam2CSK4, and the increase was evident 2 and 4 h after stimulation (Fig. 7c). Taken together, these results indicate that phagocytosis enhances Pam2CSK4-induced pro-inflammatory responses mediated by TLR2/6 signaling.

## Discussion

In this study, we observed that phagocytosis of germ-free microparticles enhances the pro-inflammatory responses of human macrophage-like cells stimulated with bacterial components including LPS and lipopeptide. Although bacterial phagocytosis is reportedly important for macrophage response, we have demonstrated that phagocytosis itself influences the responsiveness of the phagocyte by using polystyrene latex beads. Various intracellular phenomena caused by phagocytosis have been proposed to exert positive effects on TLR signaling. Moreover, the uptake of bacterial components, including LPS and lipopeptide, has been thought to occur in association with phagocytosis, which leads to the enhancement of macrophage pro-inflammatory responses. In our experiments, bead phagocytosis caused the expression of cytokines to increase beyond the maximum level of cytokine expression stimulated by LPS or lipopeptide alone. This means that phagocytosis has an effect beyond ligand enrichment. Phagocytosis of particles is known to induce polarization of macrophages, such as the M1 and M2 phenotypes^23^. However, the enhanced effects of phagocytosis in our study do not seem to be a result of macrophage polarization; THP-1 cells polarize into the M1 or M2 phenotypes after treatment for 24–48 h with various stimuli^24^, but we treated cells simultaneously with beads and TLR ligands. Therefore, we believe that the phagocytosis signal acts either synergistically or additively on the ligand signal.

There are some precedents that indicate crosstalk between TLR and the Fcγ receptor. Immune cells seeded on IgG-coated plate responded much more to LPS and/or lipopeptide than those plated on non-coated plate^25,26^. In the present study, crosstalk between TLR and the Fcγ receptor does not explain the enhancement of TLR signaling by phagocytosis since phagocytosis of PLL-coated beads is not mediated by the Fcγ receptor. The cellular receptor for phagocytosis of PLL-coated particles has not been identified, but contact with polystyrene latex beads to cell membrane activates intracellular signaling that induces actin reorganization for phagocytosis of the beads^27^. Although intracellular signaling for phagocytosis differs depending on the mechanism of particle recognition^10,27,28^, actin reorganization may be induced in cells seeded on IgG-coated plates as well as in cells that phagocytize beads^29^. Therefore, actin reorganization may be involved in enhancing TLR signaling by phagocytosis. In addition, particle size is known to affect the processes of phagocytosis such as the uptake of particles and their intracellular trafficking^30^. Use of particles with various mean diameters may be valuable for the machinery for which phagocytosis influences LPS signaling.

In this study, phagocytosis enhanced both TLR4 and TLR2/6 signaling. TLR4 activates both the myeloid differentiation primary response 88 (MyD88) pathway and the TIR domain containing adaptor inducing interferon β (TRIF) pathway whereas TLR2/6 only activates the MyD88 pathway^31–34^. Since phagocytosis enhances both TLR4 signaling and TLR2/6 signaling, phagocytosis has been proposed to enhance the common signaling pathway between TLR4 signaling and TLR2/6 signaling including the MyD88 pathway. The MyD88 pathway is the adaptor for downstream signaling by almost all TLRs^35–37^. Phagocytosis enhanced LPS-induced nuclear translocation of p65/p50 but minimally promoted IκB-α degradation. Although further analysis is required for understanding the mechanism that enhances p65/p50 activation by phagocytosis, phagocytosis seems to influence MyD88 signaling. If the phagocytosis signal acts on the MyD88 pathway, phagocytosis may have positive effects on signaling induced by almost all TLRs. However, the effects of phagocytosis on LPS-induced pro-inflammatory cytokines differed among cytokines. Phagocytosis induced robust expression of TNFα and IL-1β, but the effect on IL-6 expression was not robust. This difference in expression level may be related to differences in transcriptional regulation among cytokine genes. Transcription of the IL-6 gene with low CpG density in its promoter requires nucleosome remodeling to make it more accessible to transcription factors including NF-κB because of the otherwise condensed chromatin structure at the promoter region^38–42^. On the other hand, the TNFα gene, given the high CpG density in its promoter, may not require nucleosome remodeling^38–42^. Other cytokine genes that require nucleosome remodeling for their transcription, such as *IL12B*, may also show similar enhanced expression patterns compared with *IL6*^40–43^.

TLR signaling plays an important role for initiating the adaptive immune response^44^. Efficient TLR signaling is important for covering the immunogenicity of non-living vaccine antigens, which are often poorly immunogenic^45^. Our findings suggest that phagocytosis increases the adjuvant activity of TLR ligands. Previously, the biological activity of endotoxin has been reported to vary depending on the preparation method that affects particle size^46^. The particle size may have affected phagocytosis. To date, the phagocytosis of particulate adjuvants, including aluminum salts, are known to activate the nucleotide-binding domain and leucine-rich-repeat-containing gene family pyrin-domain containing 3 (NLRP3) inflammasome, which processes pro-IL-1β and converts it into the mature form^47,48^. Combinatorial use of particulate adjuvants and TLR ligands may be useful for inducing potent immunogenicity^49,50^. For instance, aluminum salts have been used in combination with monophosphoryl lipid A^51–53^, a detoxified derivative of LPS^54–56^. Our findings suggest that particulate adjuvants can enhance TLR signaling in addition to the NLRP3 inflammasome, and demonstrate the effects of phagocytosis itself on TLR signaling. Combinatorial use of microparticle phagocytosis should be useful for many medical applications, such as regulating inflammation, potentiating the immune system, and vaccination. In this study we analyzed effects of phagocytosis on U937 and THP-1 cell lines, both of which were human monocytic cell lines. Both cell lines differentiated into macrophage-like cells exhibited increased responsiveness to LPS due to phagocytosis of microparticles. Therefore, we speculated that some types of macrophages should increase their responsiveness to LPS by phagocytosis under certain physiological conditions; however, the types of macrophages that show similar responses have not yet been determined. Further analysis to address this issue is important not only for understanding the immune system but also for the clinical application of this study.

## Methods

### Reagents

RPMI1640 medium, polystyrene latex beads dyed blue with a mean diameter of 0.8 μm, human IgG, Cytochalasin D, poly-l-lysine (PLL), and poly (deoxyinosinic-deoxycitidylic) acid sodium salt were purchased from Sigma-Aldrich (St. Louis, MO, USA). 1α, 25-dihydroxyvitamin D_3_ was purchased from Enzo Life Sciences (Farmingdale, NY, USA). Carboxylated polystyrene latex beads dyed blue with a mean diameter of 1.0 μm were purchased from Polysciences, Inc. (Warrington, PA, USA). Penicillin–streptomycin solution, new born calf serum (NCS), monoclonal antibodies against TNFα, IL-1β, and IL-6, and biotinylated monoclonal antibodies against TNFα, IL-1β, and IL-6, were purchased from Thermo Fisher Scientific (Waltham, MA, USA). Antibodies against NF-κB subunits (p65, RelB, c-Rel, p50, and p52) were purchased from Santa Cruz Biotechnology, Inc. (Dallas, TX, USA). Anti-IκB-α antibody was purchased from Cell Signaling Technology, Inc. (Danvers, MA, USA). Secondary antibody conjugated to horseradish peroxidase was purchased from GE Healthcare Life Sciences (Chicago, IL, USA). Recombinant human TNFα, IL-1β, and IL-6 were purchased from PeproTech, Inc. (Rocky Hill, NJ, USA). LPS from *E. coli* O111:B4 was purchased from List Biological Laboratories (Campbell, CA, USA). Palmitoyl-2-cysteine-serine-lysine-4 **(**Pam2CSK4) was purchased from InvivoGen (San Diego, CA, USA). NF-κB specific oligonucleotide (Sense, 5′-AGTTGAGGGGACTTTCCCAGGC-3′) end-labeled with 6-carboxyfluorescein (FAM) was synthesized by FASMAC Corporation (Atsugi, Japan)^21^.

### Cell culture

U937 and THP-1 cells were cultured in RPMI1640 medium supplemented with 10% (v/v) heat-inactivated fetal bovine serum, 100 units/ml penicillin, and 100 µg/ml streptomycin under a humidified atmosphere with 5% CO_2_ at 37 °C. To differentiate U937 and THP-1 cells into active macrophage-like cells, the cells were resuspended in culture medium containing 0.1 μM 1α, 25-dihydroxyvitamin D_3_ to a density of 2.5 × 10^5^ cells/ml. Resuspended cells were incubated at 37 °C for 3 d^57,58^. After differentiation, medium containing 1α, 25-dihydroxyvitamin D_3_ was replaced with fresh culture medium, and the cells were used for analysis.

### Treatment of polystyrene latex beads with human IgG or PLL

Beads were coated with IgG at the time of use. Polystyrene latex beads dyed blue with a mean diameter of 0.8 μm were suspended in water at 0.83% (w/w), and 750 μl of the suspension was centrifuged at 15,000 *g* for 10 min at 4 °C; the supernatant was subsequently removed. Precipitated beads were resuspended in 1 ml water before they were collected again by centrifugation. The procedure for washing with water was repeated three times. Number of the beads was calculated by equation provided by manufacture, and 3.57 × 10^9^ beads were incubated in 400 µl RPMI1640 medium containing human IgG at 37 °C for 1 h. The supernatant was subsequently removed after centrifugation at 15,000 *g* for 5 min at 4 °C; the precipitated beads were suspended in 1 ml phosphate buffered saline (PBS) and the beads were collected by centrifugation at 15,000 *g* for 5 min at 4 °C. This procedure for washing with PBS was performed three times. Beads were resuspended in PBS at 8.93 × 10^10^ beads/ml. PLL (300 µg/ml) was used to coat carboxylated polystyrene latex beads dyed blue with a mean diameter of 1.0 μm by covalent coupling using the carbodiimide method according to the manufacturer’s protocol (Polysciences, Inc.). Prepared PLL-coated beads were stored at 4 °C until use.

### Pre-treatment of cells with IgG and cytochalasin D for measuring phagocytosis

Human IgG dissolved in 0.85% (w/v) NaCl solution was added to cells at final concentrations of 10 or 100 μg/ml 4 h prior to the addition of beads. Cytochalasin D dissolved in dimethylsulfoxide (DMSO) was added to cells to final concentrations of either 1 or 10 μM 1 h prior to addition of beads. The vehicle for each inhibitor was added to experimental controls. Cells were incubated at 37 ˚C until beads were added. Subsequent treatment of cells with beads was performed in the continuous presence of each inhibitor.

### Evaluation of the number of phagocytized beads

The number of phagocytized beads was evaluated based on previously published methods, with slight modifications^57,59^. All subsequent manipulations were carried out at 4 °C or on ice unless otherwise noted. The cell suspension (1 ml) was layered over 1 ml of 100% NCS in a microcentrifuge tube. The layered cell suspension was centrifuged at 100 *g* for 10 min to separate cells from free beads, and the supernatant was discarded. The precipitated cells were resuspended in 1 ml PBS, and the cell suspension was layered over 1 ml of 100% NCS again. After centrifugation, the supernatant was removed. The procedure for separating cells from free beads using NCS was performed four times in total. Next, the separated cells were resuspended in 1 ml PBS and washed twice by centrifuging at 800 *g* for 10 min, and the washed cells were lysed with 500 µl 0.1% SDS solution at room temperature to release the beads from cells. The number of beads was evaluated at an optical density of 665 nm (OD_665_) for cell lysates, since the blue beads show maximum absorbance at 665 nm^59^. Simultaneously, the protein content in cell lysates was quantified using Pierce BCA protein Assay kit (Thermo Fisher Scientific). The number of phagocytized beads was evaluated by calculating OD_665_ per 1 mg of cell protein. In addition, the number of phagocytized beads per cell was calculated using the standard curve of serial dilutions of a bead suspension.

### Determination of cytokine expression by enzyme-linked immuno-sorbent assay (ELISA)

Cell culture supernatants after 4, 8, and 24 h of stimulation were subjected to ELISA to measure the amounts of TNFα, IL-6, and IL-1β, respectively. Individual wells of an Immulon 2HB flat bottom 96-well microtiter plate (Thermo Fisher Scientific, Inc.) were coated with 2.0 μg/ml (IL-1β and IL-6) or 4.0 μg/ml (TNFα) monoclonal antibody (either 50 or 100 µl) at 4 °C overnight. After that, the wells were blocked with 200 µl of either 5% or 10% (w/v) skimmed milk in either PBS or PBS containing 0.2% (v/v) Tween 20 at room temperature for 1 h. Subsequently, 50 µl of culture supernatant was added to each well and mixed with 50 µl of biotinylated monoclonal antibody at a final concentration of either 0.1 μg/ml (TNFα and IL-6) or 0.25 μg/ml (IL-1β). Recombinant forms of each cytokine were used as the standard. Next, the plate was gently shaken at room temperature for 2 h. Biotinylated antibody was detected using an avidin–biotin system using VECTASTAIN Elite ABC kit (Vector laboratories, Inc., Burlingame CA, USA) and Vector 3′,5,5′-tetramethylbenzidine Peroxidase Substrate kit (Vector laboratories, Inc.). The color reaction with peroxidase was stopped by adding 100 µl 1 N sulfuric acid. Absorbance at 450 nm was measured using a microplate reader, Model 680 (BioRad Laboratories, Inc., Hercules, CA, USA).

### Preparation of nuclear extracts and cytoplasmic fraction

Nuclear extracts were prepared based on the methods published previously with slight modifications^60,61^. Briefly, 2.0 × 10^6^ cells were resuspended in 100 µl 10 mM HEPES/Na (pH 7.9) containing 10 mM KCl, 0.1 mM EDTA, 0.1 mM EGTA, 1 mM DTT, and 0.1 mM PMSF, and kept on ice for 15 min. After adding Nonidet P-40 to the cell suspension to 0.6% (v/v) and mixing vigorously for 10 s, the mixture was centrifuged at 20,400 *g* for 1 min at 4 °C. The supernatant was collected as the cytoplasmic fraction. The pellet was suspended in 20 µl of 20 mM HEPES/Na (pH 7.9) containing 0.4 M NaCl, 1 mM EDTA, 1 mM EGTA, 0.5 mM DTT, and 0.1 mM PMSF, by vigorous mixing for 15 min at 4 °C. After centrifugation of the suspension of the pellet at 20,400 *g* for 5 min at 4 °C, the supernatant was collected as the nuclear extract.

### Electrophoretic mobility shift assay (EMSA) and supershift assay

EMSA was performed based on the methods published previously with slight modifications^62^. Nuclear extract (20 μg of protein amount) was incubated with 20 µl 0.01 μM NF-κB-specific oligonucleotide as described above in 5 mM Tris–HCl (pH 8.0) containing 50 mM NaCl, 0.05% Nonidet P-40, 1 mM MgCl_2_, 0.5 mM DTT, 5% (v/v) glycerol, 0.25 mM EDTA, 40 ng/ml BSA, and 0.2 mg/ml poly (deoxyinosinic-deoxycitidylic) acid sodium salt for 30 min at room temperature. Subsequently, the reaction mixture (15 µl) was loaded on 6% native polyacrylamide gels in 1 × Tris–borate-EDTA buffer (Nacalai Tesque, Kyoto, Japan). Electrophoresis was performed at 150 V for 60 min at 4 °C. Bands of 6-FAM-labeled DNA on the gels were detected with FLA-5100 (FUJIFILM Corporation, Tokyo, Japan) using an excitation wavelength of 473 nm and emission wavelength of 510 nm. Supershift assay was performed using antibodies against NF-κB subunits: p65, RelB, c-Rel, p50, and p52. Nuclear extract (20 μg of protein amount) was incubated with 5 μg of each antibody for 30 min at room temperature in a total volume of 10 µl. Vehicle control was incubated with PBS containing 0.1% (w/v) NaN_3_. EMSA was performed as described above except that the electrophoresis was performed for 70 min.

### Western blot

Proteins (50 µg) of cytoplasmic fractions were separated by sodium dodecyl sulfate–polyacrylamide electrophoresis and electroblotted on to a nitrocellulose membrane. After blocking with 5% (w/v) skimmed milk in tris-buffered saline (TBS) containing 0.1% (v/v) Tween 20 at room temperature for 1 h, the membrane was incubated with 1/1000 dilution of anti-IκB-α antibody in 5% (w/v) BSA in TBS-containing 0.1% (v/v) Tween 20 at 4 °C overnight. Horseradish peroxidase-conjugated antibody was used to develop the membrane and visualization of bands was performed using SuperSignal West Femto Maximum Sensitivity Substrate (Thermo Fisher Scientific, Inc.) with Amersharm Imager 600 (GE Healthcare Life Sciences).

### Statistical analysis

Statistical significance was determined by Student’s t-test using Statcel 3 (OMS publishing, Tokyo, Japan), where *P* < 0.05 was considered significant. If F-test did not assume the homogeneity of variance, Welch’s t-test was used instead of Student’s t-test.

## Supplementary information

Supplementary information.
