# Supplementary material for: Phagocytosis of microparticles increases responsiveness of macrophage-like cell lines U937 and THP-1 to bacterial lipopolysaccharide and lipopeptide
Source: Sci Rep. 2021 Mar 24;11:6782. doi: 10.1038/s41598-021-86202-5 (PMC7990916; doi:10.1038/s41598-021-86202-5)
Supplement: Supplementary file 1 — Supplementary information. [file 41598_2021_86202_MOESM1_ESM.pdf]

Phagocytosis of microparticles increases responsiveness of macrophage-like cell lines U937 and THP-1 to bacterial lipopolysaccharide and lipopeptide

Takayuki Ueno, Yumi Yamamoto, and Kiyoshi Kawasaki*

*Corresponding author

Supplementary Information

Cropped images of electrophoretic mobility shift assay in Figs.6a-d, and 7c were prepared from full-length gels indicated in Supplementary Figure 1a-e, respectively. Free probes went out of the gels because of a long electrophoresis time (60~70 min) to improve separation of shifted bands (Supplementary Figure 1a-e). Free probes were observed in a gel when electrophoresis time was 35 min (Supplementary Figure 1f, samples were prepared same as Supplementary Figure 1a). A cropped image of western blot in Fig.6e was prepared from a full-length blot indicated in Supplementary Figure 1g. Dotted squares represent cropped images.

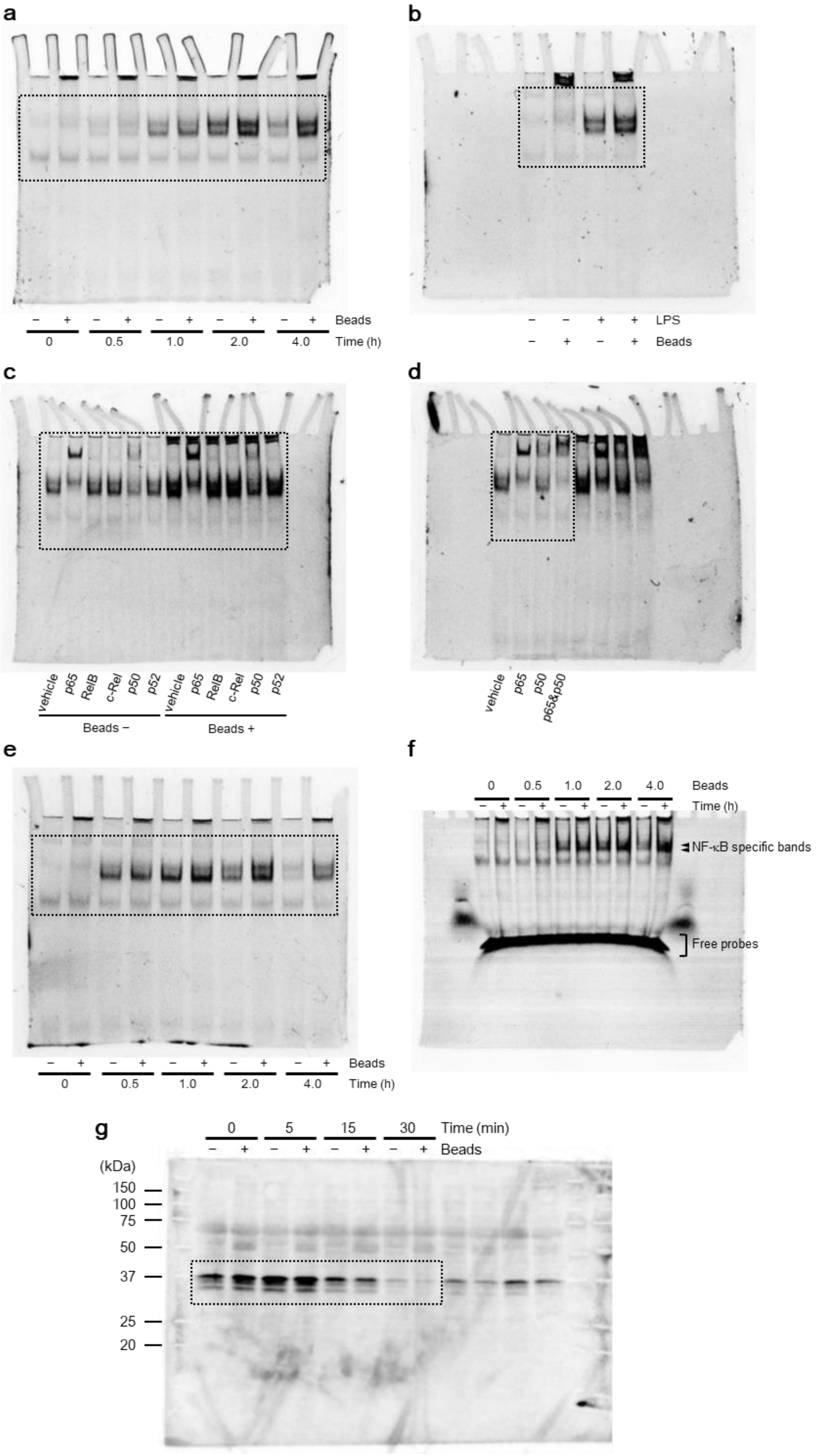

Supplementary Figure 1. Full length gels for electrophoretic mobility shift assay and a full-length western blot.
